# Poxviruses Utilize Multiple Strategies to Inhibit Apoptosis

**DOI:** 10.3390/v9080215

**Published:** 2017-08-08

**Authors:** Daniel Brian Nichols, William De Martini, Jessica Cottrell

**Affiliations:** Department of Biological Sciences, Seton Hall University, South Orange, NJ 07039, USA; william.demartini@student.shu.edu (W.D.M.); jessica.cottrell@shu.edu (J.C.)

**Keywords:** poxvirus, Vaccinia Virus, Molluscum Contagiosum Virus, Myxoma Virus, apoptosis, immune evasion, mitochondrial membrane permeabilization, protein kinase R, caspase, host defense

## Abstract

Cells have multiple means to induce apoptosis in response to viral infection. Poxviruses must prevent activation of cellular apoptosis to ensure successful replication. These viruses devote a substantial portion of their genome to immune evasion. Many of these immune evasion products expressed during infection antagonize cellular apoptotic pathways. Poxvirus products target multiple points in both the extrinsic and intrinsic apoptotic pathways, thereby mitigating apoptosis during infection. Interestingly, recent evidence indicates that poxviruses also hijack cellular means of eliminating apoptotic bodies as a means to spread cell to cell through a process called apoptotic mimicry. Poxviruses are the causative agent of many human and veterinary diseases. Further, there is substantial interest in developing these viruses as vectors for a variety of uses including vaccine delivery and as oncolytic viruses to treat certain human cancers. Therefore, an understanding of the molecular mechanisms through which poxviruses regulate the cellular apoptotic pathways remains a top research priority. In this review, we consider anti-apoptotic strategies of poxviruses focusing on three relevant poxvirus genera: *Orthopoxvirus*, *Molluscipoxvirus*, and *Leporipoxvirus*. All three genera express multiple products to inhibit both extrinsic and intrinsic apoptotic pathways with many of these products required for virulence.

## 1. Introduction

### 1.1. Overview of Apoptotic Signaling Pathways

**Figure 1:**
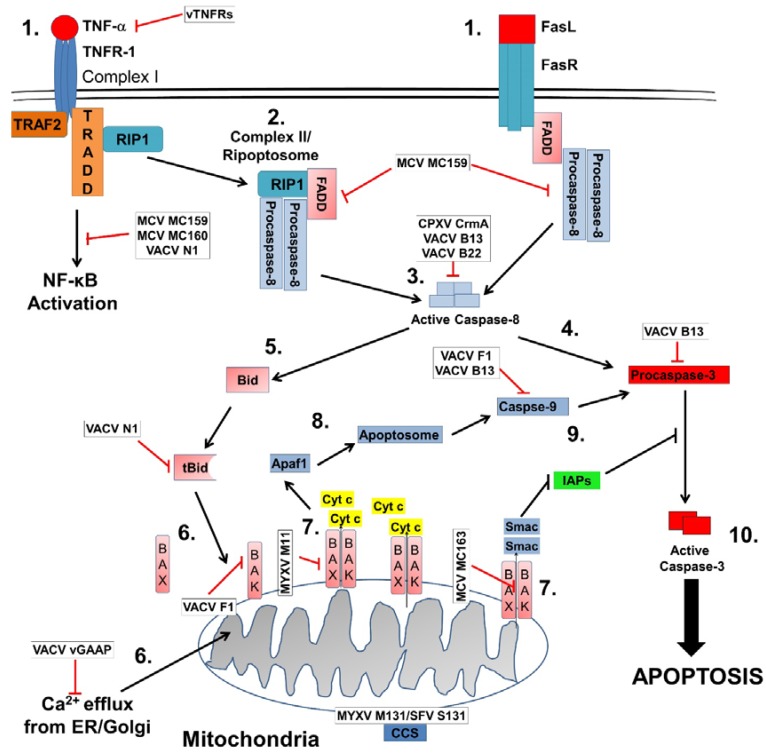
Overview of the extrinsic and intrinsic apoptotic pathways. (**1**) Tumor necrosis factor α (TNFα) or the Fas ligand (FasL) bind to the respective TNF-receptor (TNFR)-1 or FasR receptors. Fas-associated death domain protein (FADD) binds to the cytoplasmic region of FasR and forms a scaffold that recruits procaspase-8.For TNF, TNFR-associated death domain protein (TRADD) associates with the cytoplasmic death domain (DD) of the TNF-R1 and forms complex 1 which leads to nuclear factor κB (NF-κB) activation; (**2**) Alternatively, TNF can induce apoptosis when receptor-interacting protein 1 (RIP1) forms a cytoplasmic complex II consisting of RIP1, FADD, and procaspase-8; (**3**) Procaspase-8 oligomerization results in its autocleavage and activation where the initiator caspase-8 activates (**4**) caspase-3 or cleave additional substrates such as (**5**) BH3 interacting-doain death agonist (Bid) to truncated (t)Bid; (**6**) tBid activates Bcl-2 homologous antagonist killer (Bak)/Bcl-2-associated X protein (Bax) oligomers in the mitochondria. Alternatively, Bak/Bax can form pores in the mitochondria outer membrane in response to Ca^2+^ efflux from the endoplasmic reticulum (ER) or Golgi; (**7**) Bax/Bak pores result in mitochondria membrane permeabilization which leads to the subsequent release of cytochrome *c* and second mitochondria-derived activator of caspases/direct inhibitor of apoptosis protein with low pI) (Smac/DIABLO, referred to as “Smac” in the illustration) from the inner membrane space of the mitochondria to the cytosol; (**8**) Cytoplasmic cytochrome *c* binds Apaf1 leading to the formation of the apoptosome and the activation of initiator caspase-9; (**9**) caspase-9 in turn activates effector caspases such as caspase-3.Smac released form the mitochondria also binds inhibitor of apoptosis proteins (IAPs) which allows caspase-3 to become active and cleave target proteins; (**10**) Effector caspases in turn cleave target proteins resulting in the activation of apoptosis. Poxvirus proteins are indicated in the open boxes. Red lines indicate points in the pathway inhibited by viral proteins. Vaccinia virus (VACV) F1, Myxoma virus (MYXV) M11, MYXV M131, Shope Fibroma Virus (SFV) S131, and Molluscum Contagiosum Virus (MCV) MC163 localize to the mitochondria where these proteins antagonize mitochondria mediated responses in the intrinsic apoptotic pathway. MYXV M131/SFV S131 are depicted interacting with cellular copper chaperones for superoxide dismutase (CCS).

Apoptosis is a conserved process that can be triggered by both extrinsic and intrinsic stimuli. In both cases, activation leads to the accumulation of a class of cysteine proteases known as caspases that are found in most cell types [1,2]. Caspases are vital to apoptosis and are more abundant in organisms with higher complexity. However, caspases do have many non-apoptotic functions in processes such as immunity, learning, and cognition [3,4,5]. Caspases are classified by function. Initiator caspases enable the formation of protein platforms that regulate caspase activation while executioner caspases help with processes after mitochondrial outer membrane permeabilization (MOMP) [6]. MOMP is integral to the decision for a cell to commit to programmed cell death (PCD). Members of the B-cell lymphoma-2 (Bcl-2) family regulate MOMP and therefore determine whether a cell will undergo apoptosis. When apoptosis is initiated, the outer mitochondrial membrane releases intermembrane proteins such as cytochrome *c* into the cytosol. This event triggers the formation of the apoptosome, activation of executioner caspases, and proteolytic cleavage of numerous crucial cellular target proteins. Eventually, this results in the inactivation of DNase inhibitors, which allows the nuclear DNA to be fragmented [6]. This process is highly regulated and varies based on the main mechanisms of induction. The mechanisms of extrinsic, intrinsic, and double stranded RNA (dsRNA) induced apoptosis are discussed below (Figure 1).

### 1.2. Extrinsic Apoptotic Pathway

Extrinsic apoptosis also known as death receptor mediated apoptosis typically involves activation of tumor necrosis factor (TNF) superfamily receptors [7]. Cytokines such as TNFα, Fas ligand (FasL), or TNF-related apoptosis-inducing ligand (TRAIL) associate with their respective receptor through an amino-terminal cysteine-rich domain (CRDs). These CRDs define their ligand specificity while a section of 60–70 amino acids known as the death domain (DD) is important for apoptosis induction [8,9]. Once a cytokine has bound to its cognate receptor, the recruitment of adaptor proteins such as Fas-associated death domain protein (FADD), TNF-receptor (TNFR)-associated death domain protein (TRADD), TNFR2-associated factor 2 (TRAF2) or receptor-interacting protein 1 (RIP1) can occur [10]. Next, these proteins assemble to form the death-inducing signaling complex (DISC). DISC provides the scaffold necessary to recruit and activate the initiator caspase, pro-caspase-8 through FADD’s death effector domain (DED) [10]. Pro-caspase-8 is activated via proteolytic cleavage and the release of its active p18/p12 domain. The liberated caspase-8 activates downstream caspases-3, -6, and -7 which participate in the execution of the apoptotic process [8,11]. Regulation over complex II also known as a ripoptosome can occur in multiple ways. FLICE (FADD-like IL-1β-converting enzyme)/caspase-8 inhibitory protein (FLIP) recruitment to the DISC can interact with caspase-8 in the complex and inhibit PCD [12]. Second, the silencer of death domain protein (SODD) can act as an intracellular inhibitor of TNFR to prevent constitutive activation [13]. Finally, cellular inhibitor of apoptosis proteins (cIAPs) can control DISC formation. cIAPs can stimulate TNFα and help mediate the activation of nuclear factor κB (NF-κB) under certain conditions [14]. Poxviruses have multiple strategies to prevent the activation of these pathways and are discussed below.

### 1.3. Intrinsic Apoptotic Pathway

The intrinsic apoptotic pathway is regulated by Bcl-2 family members which work together to control the integrity of the outer mitochondrial membrane (OMM). During apoptosis, functionally redundant Bcl-2 family members such as Bcl-2-associated x protein (Bax) and Bcl-2 antagonist killer 1 (Bak) converge to direct mitochondrial membrane permeabilization (MMP) [15], which allows soluble mitochondrial proteins like cytochrome *c* to leak to the cytosol. MOMP also allows the release of the second mitochondria-derived activator of caspases/direct inhibitor of apoptosis protein with low isoelectric point (pI) (Smac/DIABLO) [6]. Smac/DIABLO inhibits cellular inhibitor of apoptosis proteins (cIAPs) thereby promoting apoptosis. Within the cytosol, cytochrome *c* and apoptotic protease factor-1 (APAF-1) engage to oligomerize into a caspase activation platform termed an apoptosome [16,17]. The apoptosome promotes the activation of initiator caspase-9, which in turn will activate executioner caspases-3 and -7. These caspases will begin a cascade of proteolytic cleavage on important cellular substrates, which will eventually lead to the nuclear DNA to be fragmented. It is important to note that the extrinsic and intrinsic pathways are not mutually exclusive with many aspects of the extrinsic pathway capable of inducing intrinsic apoptosis through the mitochondria. For example, active caspase-8 cleaves Bid into tBid. tBid in turn induces the formation of Bax/Bak oligomers within the OMM [6].

Bcl-2 protein function is conserved in vertebrates and limited data exists for function in invertebrates [18]. These proteins are globular, α-helical, and classified according to their anti-apoptotic or pro-apoptotic functions. All Bcl-2 proteins share conserved homology domains known as Bcl-2 homology (BH) regions [17]. Even close family relatives like Bcl-XL have BH regions. The anti-apoptotic family members include Bcl-2, Bcl-x, Mcl-1, and A1 and possess four conserved BH regions known as BH1-4. Pro-apoptotic family members are divided into two classes: “multidomain” which contain BH1-3 domains or “BH3-only” proteins. Bak, Bax, and Bcl-2 related ovarian killer (Bok) are mutlidomain family members while Bcl-2 like protein 11 (Bim), Bid, p53 upregulated modulator of apoptosis (PUMA), and Noxa are BH3-only family members. The regulation of OMM by Bcl2 family members is still unclear. However, direct activation, displacement, and embedding together are current models of the Bcl-2 family’s role during apoptosis. Each model describes critical protein-protein and protein-membrane interactions of the Bcl-2 family during apoptosis and is extensively reviewed by Leber et al. [15]. In brief, BH3-only proteins, Bim and Bid are thought to act upstream of the multidomain proteins Bak and Bax to initiate apoptosis through direct binding interactions [19]. Bak and Bax then homo-oligomerize into proteolipid pores within the OMM [20]. The resulting pore formation releases intermembrane space proteins, which interact with various cellular proteins. These proteins activate proteases, caspases, and nucleases that will physically destroy the cell. The Bcl-2 family is conserved among vertebrates. Dynamic organelles such as mitochondria undergo fission and fusion regularly to yield an interconnected tubular mitochondrial network [16]. Cytokines and GTPases regulate these processes. Often MOMP coincides with fragmentation of the mitochondrial network suggesting the Bcl-2 proteins have important role in modulating the balance between fission and fusion [21]. The role of Bax and Bak in MOMP has been shown to be important but a precise mechanism is unclear [22,23,24,25]. After pro-apoptotic stimulation, mitochondrial fusion and fission machinery components are recruited to the scission sites and colocalize with Bax [26]. Studies showed that the process of MOMP and fission are linked [26,27,28]. However, several studies suggest that these processes are not interdependent on each other [29,30,31,32,33]. Cytochrome *c* cannot diffuse freely within the inner mitochondrial membrane (IMM). However, caspase dependent cristae rearrangements can occur during MOMP which allow cytochrome c to freely diffuse out of the IMM [34,35].

The mitochondria and the endoplasmic reticulum (ER) also interact during apoptosis.ER calcium Ca^2+^ levels can be influenced by the Bcl-2 family effecting storage and signaling [17]. Rong et al. demonstrated that ER-localized Bcl-2 and its BH4 domain can directly inhibit inositol 1,4,5-triphosphate (IP3) receptor on the ER [36]. The IP3 receptor is an IP3-gated Ca^2+^ channel that regulates many processes including cell proliferation and death [36]. In a mouse embryonic fibroblast model, multidomain proteins Bak and Bax were found to dysregulated Ca^2+^ uptake in the ER and mitochondria. Dysregulated Ca^2+^ levels inhibited apoptotic death, which was restored by introducing corrected calcium levels within the model [37]. BH3 domains like Bim and PUMA are also known to induce ER Ca^2+^ release; however, the mechanism by which this occurs is unknown [38]. Interestingly, DNA microarray analysis found PUMA was upregulated by ER stress. After a global RNA interference screen, PUMA was found to have a functional role in ER stress-mediated apoptosis [39,40]. Finally, dysregulated Ca^2+^ homeostasis can lead to unfolded protein response (UPR). UPR can compromise the ER functions of protein modification, folding, and secretion [17]. Bcl-2 family members such as Bak and Bax can help the ER recover from UPR by directly binding and regulating inositol requiring enzyme (IRE1) ER signaling.

The ER is the primarily recognized as the site of protein synthesis and folding of secreted, membrane-bound, and organelle targeted proteins. Proper ER function requires optimum levels of ATP and Ca^2+^ as well as an oxidizing environment conducive to disulphide-bond formation [41]. Cellular stress effecting energy levels, the redox state or Ca^2+^ concentration greatly reduce the folding capacity of the ER. As the folding capacity of the ER decreases, unfolded proteins aggregate and accumulate resulting in ER stress. Protein aggregation is toxic to cells and can lead to disease [42]. In response to ER stress, the unfolded protein response (UPR) can be activated by three ER transmembrane receptors: pancreatic ER kinase-like ER kinase (PERK), activating transcription factor 6 (ATF6), and IRE1. When unfolded proteins accumulate, GRP78, an ER chaperone, will dissociate from these receptors and trigger the unfolded protein response (UPR). The UPR response is typically a prosurvival response that functions to reduce the accumulation of unfolded proteins in the ER [43,44]. However, when protein accumulation becomes persistent it switches from a prosurvival signal to a pro-apoptotic signal. Signaling through PERK, ATF6, and IRE1 do not directly cause PCD but activate CCAAT-enhancer-binding protein (C/EBP) homologous protein (CHOP) and c-Jun N-terminal kinase (JNK) pathway which promote cell death [45,46]. CHOP has been found to interact with Bcl-2 proteins, which can promote or enhance apoptotic activation [47]. JNK can also regulate Bcl-2 by phosphorylation. Phosphorylated Bcl-2 is unable to sequester and inhibit pro-apoptotic BH3-only proteins and cannot control ER Ca^2+^ fluctuations [48]. ER stress induced JNK activation can target Bcl-2 proteins and lead to the activation of Bax and Bak proteins leading to the execution of apoptosis [49,50]. The caspases thought to be involved in ER stress include caspases 12, 3, 6, 7, 8, and 9. Currently, caspase 12 has been proposed to be the initiator caspase in ER stress-induced apoptosis but the evidence supporting this claim is still uncertain [51,52,53].

### 1.4. dsRNA Induced Apoptosis

Double stranded (ds)RNA has a well-established role in activating anti-viral responses through the upregulation of type I interferon (IFN-1) [54]. dsRNA has also been shown to play a role in caspase-8-dependent apoptosis [55,56]. The toll-like receptor 3 (TLR3) and the cytosolic protein kinase RNA-activated (PKR) are thought to trigger IFN-1 production and lead to PCD [55]. dsRNA released during a viral infection can stimulate the pattern-recognition receptor TLR3. When dsRNA binds to the TLR3, dimerization occurs, and the Toll/interleukin (IL)-1 receptor (TIR) cytoplasmic domain reorients, and enables the recruitment of the adapter molecule TIR domain-containing adapter inducing IFN_2_ (TRIF).TRIF then recruits TNF receptor-associated factor (TRAF)-6 and the RIP-1 serine-threonine kinase that activates nuclear factor-κB (NF-κB) and pro-apoptotic gene expression. Alternatively or in combination with the above mechanism, TRIF can recruit TRAF-6, RIP-1, and TRAF3 and activate the IFN regulatory factor 3 (IRF-3) and the downstream IFN response [57]. TLR3 can induce apoptosis via caspase-8 even though it lacks a death domain [55,58,59,60,61]. Although TLR3’s role in this signaling complex remains unclear, one study suggests that the C-terminal RIP homotypic interaction motif (RHIM) of RIP1 interacts with TRIF to trigger caspase-8 induced apoptosis [62]. Other data has shown that cIAPs negatively regulate TLR3-induced apoptosis [55,56,58,63]. Therefore, data-providing information about the molecular assembly of this TLR-3 inducing apoptosis complex is still needed.

Double stranded (ds)RNA can also induce IFN through the serine-threonine protein kinase PKR. The protein domains of PKR can be linked to the α subunit of eukaryotic translation initiation factor 2 (eIF-2α) family and the dsRNA-binding protein family [64]. When dsRNA induces IFN expression, PKR expression is stimulated. PKR inhibits protein synthesis through the phosphorylation of its cellular substrate eIF-2α which prevents the regeneration of the GTP in the ternary complex eIF2α-GTP-tRNA^Met^ [65]. Typically eIF-2α phosphorylation negatively regulates ribosome scanning and/or the direct attachment of internal ribosomal entry sites (IRES) [66] and leads to apoptosis. A number of viruses including poxviruses have evolved strategies to escape this control mechanism [67,68]. Data suggests that another cellular substrate of PKR, inhibitor of nuclear factor κ-B kinase subunit (IκB) α can participate in the regulation of this apoptotic pathway [69,70,71]. In many cells, heterodimers of NF-κB associate with IκBα proteins and render it inactive. However, when responding to activators, IκBα, can be phosphorylated. Once released from NF-κB, the IκBα’s undergo proteolysis via the ubiquitin-proteasome pathway [72]. With the loss of these IkBs and the unmasking of its nuclear localization signal, NF-κB can translocate to the nucleus and upregulate the transcription of target genes [70]. TRAF proteins may help recruit the IκB kinase to exert control of the PKR induced apoptosis [73,74].

### 1.5. Poxvirus Background

Poxviruses have significant relevance to public health. For example, the Molluscum Contagiosum Virus (MCV) causes a common skin infection in humans resulting in persistent lesions that are difficult to control especially in immunocompromised individuals [75]. Emerging zoonotic diseases such as Monkeypox (MPXV) and Vaccinia Virus (VACV) infections continue to impact human health in infected areas [76,77]. Further, because the human population is no longer vaccinated, the intentional or unintentional release of Variola Virus (VARV), the causative agent of smallpox, remains a concern. Poxviruses also represent attractive vectors with many medically relevantuses ranging from vaccines to use as oncolytic viruses [78,79,80]. Therefore, understanding how these viruses modulate host immune responses, including cellular apoptotic pathways, remains a research priority.

Poxviruses are linear, dsDNA viruses that replicate exclusively in the cytoplasm of the host cell. These complex DNA viruses have large genomes of approximately 130–375 kbp that encode for numerous virus proteins [81]. Poxviruses have a remarkably complex virus life cycle. Upon entering a host cell, the virus transcribes the genome in a temporally regulated cascade of early, intermediate, and late genes. Early gene transcription occurs in the nucleocapsid with early transcription factors already bound to early promoters, and early messages are extruded through pores into the cytoplasm [81]. Given the complex nature of poxvirus replication, these viruses are exposed to an array of cellular proteins that detect and respond to infection. Many poxvirus early genes encode products with immune evasion functions [11,82,83]. In addition, some late transcription products with immunomodulatory function are packaged in viral particles called lateral bodies [84,85]. Presumably, these products are then delivered to the host cell and allow the virus to immediately begin subverting the host cell’s innate immune responses. In all, about one third to one half of the poxvirus genome is devoted to immune evasion [82]. Many of these poxviral immune modulatory proteins are required for pathogenicity and to confer host range [84]. Given that the cell has multiple pathways to induce apoptosis and other cell death pathways (Reviewed in [11]) during a virus infection, it is not surprising that multiple poxvirus products are involved in antagonizing the cell’s apoptotic response, thus allowing the virus to complete its life cycle and produce progeny virions (Table 1). While some of these viral proteins functions have been elucidated, the majority remain unknown. The study in how these viral products elicit their function in host cells has provided significant insights as to how viruses interact with host cells and how host cells sense and respond to virus infection. Here, we review anti-apoptotic strategies of several important poxviruses.

## 2. Poxvirus Inhibitors of the Extrinsic Pathway

### 2.1. TNF Receptor Orthologs

Decoy receptors are used by poxviruses to antagonize the host’s ability to respond to infection. Through the inhibition of cytokines such as TNF binding to its receptor, poxviruses attenuate both inflammation and apoptosis. The TNF superfamily is composed of 19 members that bind to 20 cellular receptors of the TNF receptor superfamily [86]. TNF is first expressed as a transmembrane cytokine where it can be processed to a soluble cytokine by the metalloprotease TNFα-converting enzyme (TACE/ADAM17) [86,87,88]. The receptors for TNF bioactivity include TNFR1 and TNFR2. Whereas TNFR1 is expressed ubiquitously with conserved death-domain motifs TNFR2 is expression is restricted to immune and endothelial cells and lacks a death domain [89]. Many poxviruses employ molecular decoys, called viral TNF receptors (vTNFR), to mitigate the effects of TNFα. The contribution of vTNFRs to the pathogenesis of poxviruses has been examined in the literature. Extracellular TNF is captured by these secreted vTNFRs which lack transmembrane and signaling domains [90].

Five different vTNFRs have been described in orthopoxviruses: cytokine response modifier B (CrmB), CrmC, CrmD, CrmE and the viral homolog of CD30 (vCD30). vCD30 is encoded in ectromelia virus and binds the CD30L preventing its interaction with CD30 [91]. vCD30 is an inhibitor of T helper cell-mediated inflammation [91]. However, vCD30 is not a major virulence factor in the mousepox model [92]. The remaining vTNFRs bind cellular TNF. CrmE has been shown to bind TNF by crystallography [93]. The vTNFRs mimic the extracellular domain of cellular TNFR1/2 on the N-terminal region. The N-terminal region of these vTNFRs contain up to four conserved TNF binding cysteine rich domains (CRDs) [93]. VARV CrmB, cowpox virus (CPXV) CrmB and ectromeliavirus CrmD have an additional C-terminal extension, which has been named smallpox virus-encoded chemokine receptor (SECRET) domain. This domain has a high affinity for certain chemokines [94].

The differences between CrmB, CrmC, CrmD and CrmE are due to ligand affinity and their expression in orthopoxviruses [95]. Certain strains of VACV (USSR, Lister and Evans) express these vTNFRs via the *crmC* and *crmE* viral genes. The majority of the strains for VACV encode gene fragments related to vTNFR but do not encode a functional protein [82]. Investigation of vTNFRs by Reading et al. indicated that CrmB, CrmC and CrmE enhance the virulence of recombinant VACV [96].

The *crmB* gene is important in the pathogenicity of various poxviruses. CPXV lacking CrmB have increased lethal dose (LD)_50_ in infected mice. In MPXV, the *crmB* gene is present in two copies and is the only vTNFR encoded in the genome [97]. In all viral species CrmB orthologs are expressed early in infection, while CPXV CrmC and CPXV CrmD are translated later in viral life cycle [98,99]. The only predicted gene to be active in VARV is the *crmB* gene [94,100]. A 2015 study by Pontejo et al. reported on the functional and binding properties of poxvirus vTNFRs [97]. CrmB from VARV is the most potent of the tested vTNFRs with a binding affinity for TNF stronger than the biopharmaceutical etancercept, a soluble form of the human TNF receptor 2 (hTNFR2) [97]. The binding affinity constant (K_d_) of VARV CrmB to human TNF is 0.28 nM whereas the K_d_ of hTNFR2 is 0.3 nM. It was observed that CPXV CrmB possesses a higher binding affinity than hTNFR2 with mouse TNF with a K_d_ of 0.12 nM and 0.43 nM respectively [97].

Additionally, vTNFRs are present in *Leporipoxvirus* Shope Fibroma Virus (SFV). T2 was the first protein identified as a vTNFR.Similar to other vTNFRs, T2 sequesters TNF resulting in the inhibition of cellular TNF receptor activation and responses from downstream antiviral processes [101,102]. In the closely related MYXV, M-T2, described as the first “viroceptor”, is an important virulence factor. Absence of M-T2 results in reduced pathogenicity in rabbit models. Two forms of M-T2 serve different functions. The secreted form of M-T2 binds and inhibits TNF while the intracellular version blocks virus induced lymphocyte apoptosis [103,104]. The anti-apoptotic function of intracellular M-T2 to inhibit TNFR1 induced cell death requires a highly conserved viral preligand assembly domain (vPLAD) located on the N-terminus [105]. These aforementioned interactions detail a fascinating mechanism by which poxviruses employ a protein decoy based defense mechanism in the continuing molecular arms race between the poxvirus and host’s innate immune response.

### 2.2. Serine Protease Inhibitors (Serpins)

Poxviruses express several proteins to antagonize the function of caspases called serine protease inhibitors (serpins). Members of the serpin superfamily consist of a single polypeptide chain (370 to 390 amino acid residues) with a conserved domain of three βsheets and nine α helices [106]. The C-terminus portion of serpins possess a specific site called the reactive-site loop (RSL) which interacts with a serine or cysteine protease by acting as a substrate mimic. The RSL site is structurally located on a distorted α helix that extends from β-Sheet A [107]. The inhibitory function of serpins is executed through forming long lasting complexes with their target proteases with their substrate stable acyl-enzyme intermediates [108]. Specificity is primarily defined in serpins by the P1 residue within the RSL. For example, the serpin CrmA in CPXV has a P1 of aspartate which directs the protein to inhibit granzyme-B (serine protease) and caspases (thiol proteases) [109,110,111]. Specific mutations critical for serpin activity are related to human disease. When the wildtype methionine in P1 is mutated to an arginine in the serpin α-antitrypsin Pittsburgh elastase, the mutant gains the ability to inhibit trypsin like enzymes causing severe bleed disorder [107,112]. Serpins are present in numerous poxviruses including *Orthopoxviruses* CPXV and VACV and *Leporipoxvirus* MYXV. Several studies have shown their role in anti-inflammatory, anti-apoptotic and virulence processes. *Orthopoxviruses* and *Leporipoxviruses* encode for three serpins [113].

The first identified serpin was CrmA from CPXV. This serpin is also known as B13R in VACV. The CrmA inhibits caspase 1 (IL-β-converting enzyme, ICE) [110,114] whose activity produces mature proinflammatory cytokines such as IL-1β from proIL-1β. The proinflammatory cytokine IL-1β is important in controlling poxvirus infections [115,116]. Palumbo et al. reported that the deletion of CrmA from CPXV produces white inflammatory lesions in embryonated chicken eggs chorioallantoic membranes (CAMs) whereas typically wild type CPXV produce red non-inflammatory lesions. The CrmA deleted CPXV lesions exhibited lower amount of CPXV virus replication compared to wildtype [117,118].

In addition to being a viral inhibitor of inflammation, the CrmA protein has anti-apoptotic properties in culture cells [119,120]. CrmA inhibits apoptosis in swine cells infected with CPXV. CrmA inhibits the activation of multiple caspases, which are crucial initiator caspases in the extrinsic and intrinsic apoptotic pathway [2,121,122,123].

The VACV protein B13 (SPI-2) shares 92% amino acid with CPXV CrmA.Not surprisingly, B13 functions very similar to CPXV CrmA. Like CrmA, B13 inhibits multiple initiator caspases and can inhibit apoptosis induced by a variety of challenges including TNFα, FasL, staurosporine, and the DNA damaging agent doxorubicin (DOX) [2,11,119,120,124]. Recently, a study by Veyer et al. compared the anti-apoptotic activity of four different VACV proteins: B13, F1, N1 and viral Golgi anti-apoptotic protein (vGAAP) [125]. The authors utilized recombinant VACV strain (vv811), which lacks 55 genes including those coding for several VACV anti-apoptotic proteins. When expressed in strain vv811, B13 was the most potent inhibitor of both the extrinsic and intrinsic apoptotic pathways [125].

Vaccinia virus B22R (SPI-1) gene encodes for a similar protein to SPI-2/(CrmA). B22R is 44% identical to B13Rwith a different reactive center [126,127]. Both serpins SPI-1 and SPI-2 are expressed early in the viral infection process and remain inside the host cell [126,127,128]. In a study by Shisler et al. the importance of SP1-1/B22 in VACV was examined by means of a mutated VACV lacking the SPI-1/B22R gene. Due to this gene depletion, viral replication in A549 cells was lowered by almost two logs in one-step growth curve. Not surprising, there was a reduction in virus particles as well intermediate and late mRNA, viral late protein and cleave proteins. A549 cells lacking the SPI-1 gene were found to be sensitive to TNF induced apoptosis [129]. The SPI-3 protein, known for inhibiting cell fusion [130] is yet another VACV serpin but is not required for virulence in VACV and CPXV in intranasal inoculated mice [131,132].

The *Leporipoxvirus* MYXV genome encodes for two intact serpins SERP1 and SERP2 and a truncated SERP3 [133]. The SERP1 serpin in MYXV is required in vivo for full virulence with mutations in both genes of SERP1 causing significant attenuation. SERP1 is a late virally expressed protein and reduces inflammation following MYXV infection [134]. Like SPI-3, SERP1 possesses an arginine residue at the P1 in the RSL and possesses a similar proteinase inhibitory profile which suggests they have similar functional in vivo [135,136]. Wang et al. investigated the phenotypic effects of swapping SPI-3 and SERP1 from their native virus genome, MYXV and CPXV respectively while not modifying the wildtype promoters in order to maintain their viral temporal expression. Despite their similarity, these two serpins are not interchangeable between MYXV and CPXV [135].

In keeping with the theme of switching serpins between different poxviruses, Nathaniel et al. studied the effect of swapping CPXV CrmA with MYXV SERP2. The serpin SERP2 possesses arginine residue at the P1 in the RSL similarly as CrmA but only shares a 35% amino acid identity but does possess similar functionality for inhibition of caspase 1 and granzyme-B. MYXV lacking the SERP2 serpin are attenuate in rabbits [137,138,139]. Despite these functional similarities, CrmA and SERP2 are not fully interchangeable. SERP2 does not inhibit inflammation, but restores viral load in CPXV infected CAMs. CrmA restores partial MYXV virulence however lesion morphology was not fully recovered [140].

SERP-3 was characterized 2001 by Guerin et al. as the third serpin in MYXV. The SERP-3 serpin has a significant amount of deletions compared to other viral serpins and does not share much amino acid identity to SERP-1 (19%) or SERP-2 (31%). SERP-3 contains several conserved motifs found commonly in serpins. *Serp3* transcripts are detectable at 8 h post infection and as late 16h using reverse transcriptase PCR in MYXV infected RK13 cells [141]. Rabbits inoculated with MYXV without the *serp3* gene produced small, thin and less congested lesion as compared to wildtype. Eight days post infection the rabbits displayed symptoms of respiratory and conjunctival bacterial infection rather than the severe symptoms found with myxomatosis [141]. Based on the aforementioned findings, serpin activity is at its full efficacy in its native virus despite the functional similarities two serpins originating from different poxviruses. The mode of action of these serpins needs to be elucidated.

### 2.3. The Molluscum Contagiosum Virus Death Effector Domain Containing Proteins MC159 and MC160

Relative to other poxviruses, MCV utilizes unique strategies to antagonize the extrinsic apoptotic pathway [83]. Many of the apoptotic modulators present in VACV are absent in the MCV genome [142,143]. MC lesions are characterized by increased hyperplasia and hypertrophy [75]. Two types of MC lesions have been described, inflamed (I-MC) and non-inflamed MC (NI-MC) [144]. Of interest, NI-MC lesions appear to have limited apoptotic responses. A study conducted by Vermi et al. found caspase-3 to be in the inactive in NI-MC lesions. However, abundant apoptotic cell death is present at the site of I-MC lesions [144]. Such observations may highlight a struggle between host innate immune responses and the ability of MCV to subvert host cell immune responses including apoptosis. Given the lack of apoptotic responses at NI-MC lesions and overall persistence of MCV infections, it is expected that MCV produce several viral proteins that dampen host cell apoptotic responses. However, due to the lack of a cell culture system or available animal model to study MCV processes, identification of MCV proteins that regulate cellular apoptosis has thus far relied on ectopic expression or use of surrogate viruses that express MCV proteins [83].

The Molluscum Contagiosum Virus MCV encodes two viral proteins, MC159 and MC160, each possessing two tandem death effector domains (DEDs) [143]. DEDs are involved in protein-protein interaction and are found in a variety of pro- and anti-apoptotic signaling molecules. MC159 and MC160 are predicted to be expressed from early gene promoters during an MCV infection [143]. Both MC159 and MC160 belong to a family of proteins collectively referred to as viral FLICE-like inhibitory proteins (vFLIPs) [83]. This family of viral proteins regulates several host pathways involved in innate immune response including pathways that lead to the activation of apoptosis, NF-κB, interferon, and necroptosis [83,145,146,147,148,149,150,151,152]. In addition to the MCV proteins, vFLIPs are also expressed by several gamma herpesviruses including human herpes virus 8 and equine herpes virus [12,153,154].

Death effector domainsare found in several pro-apoptotic host proteins including FADD and procaspase-8. FADD possesses a single DED while procaspase-8 contains two tandem DEDs, similar in arrangement to the DEDs of MC159 and MC160 [83,155,156,157]. During activation of the extrinsic pathway by inducers such as FasL, FADD and procaspase-8 assemble through DED interactions at the receptor to form the DISC [158]. Upon interaction with FADD, procaspase-8 in turn forms oligomeric filaments consisting of many molecules of procaspase-8 [159,160,161,162]. The procaspase-8 self-association induces its autocleavage into the active effector caspase-8, which in turn cleaves pro-apoptotic products leading to activation of the extrinsic apoptotic pathway [163]. The cellular FLICE-inhibitory protein (cFLIP) also contains two tandem death effector domains and is capable of regulating apoptosis upon association with procaspase-8 [164,165].

Of the two MCV DED-containing proteins, the MC159 protein has been the best characterized in terms of its role during apoptotic signaling. The MC159 protein is comprised of 241 amino acids with the two tandem DEDs located at the N-terminus [83]. The crystal structure of MC159 was one of the first DED-containing proteins to be solved. Structural analysis revealed that the MC159 DEDs tightly associate in a dumbbell conformation via hydrophobic interactions between the two DEDs [166,167]. Both MC159 DEDs contain a conserved RxDL motif (69–72 in DED1 and 166–169 in DED2). The RxDL motif is conserved in several DED-containing proteins including FADD, procaspase-8, and the homologous MC160 protein. The arginine and aspartate residues present in the RxDL motif interact with upstream glutamates in their respective DEDs (E24 inDED1 and E111 in DED2) to form a network of hydrogen bonds collectively referred to as the charge triad [166,167]. This interaction provides a key element in DED-folding and orients the side chains of adjacent amino acid residues involved in protein-protein interactions. Alanine substitutions at any of these key residues disrupt MC159 function [166,168].

MC159 expression inhibits apoptosis induced by TNFα and FasL [12,153,155,168]. Through DED interactions, MC159 associates with both FADD and procaspase-8 [153,155]. By interacting with both FADD and procaspase-8 DEDs, MC159 expression blocks the formation of death effector filaments and caps these filaments thereby blocking caspase-8 activation [162,169]. Several MC159 point mutations have been identified that lose the ability to inhibit apoptosis [168]. Many of these loss of function mutations were the result of replacing charged amino acids in or nearby the charge triad of DED1 with alanine substitutions (R69A, D71A; E18A, E19A, D21A). These MC159 mutants no longer inhibit the formation of DED-filaments in cells stimulated with Fas [168]. Further, a study by Yang et al., found that several of these MC159 mutants that could no longer inhibit apoptosis correlated with a loss of the ability to form a ternary complex with Fas/FADD [166]. Fu et al. recently published a study using cryoelectron microscopy to determine the filament structure of oligomerized caspase-8 DEDs [162]. MC159 interacts with filamentous FADD and caspase-8 to prevent further caspase-8 oligomerization. Thus, by MC159 binding and capping caspase-8 oligomers, caspase-8 activation is prevented [162]. Interestingly, the capping mechanism of MC159 is unique when compared to cFLIP. cFLIP binds to the DISC dependent on the recruitment of FADD and procaspase-8.Fu et al. present a model where cFLIP assembles with caspase-8 during oligomerization [162]. Thus, the presence of cFLIP in the oligomers likely reduces the activation of caspase-8, thereby preventing apoptosis [162].

Of note, the C-terminal portion of MC159 also possesses three TRAF3 binding motifs (PxQxS/T), which mediate the MC159-TRAF3 interaction [152]. MC159 recruits TRAF2 and TRAF3 into DISC complexes. Further, MC159 mutants lacking TRAF-binding motifs only partially protect Jurkat cells from Fas-induced cell death. Therefore, MC159 utilizes both TRAF-dependent and TRAF-independent mechanisms to prevent Fas-induced cell death [152]. However, the TRAF-dependent mechanisms are not currently well understood.

A significant interest in the field is to determine how MC159 contributes to pathogenicity during an MCV infection. Unfortunately, the lack of a suitable system to study MC159 in the context of an MCV infection is not available. Several studies have used surrogate viruses to express MC159. Shisler and Moss utilized a recombinant VACV with a *crmA* deletion expressing MC159 [155]. Relative to the parental VACV, the recombinant MC159 virus prevents Fas-induced activation of caspase-3 and caspase-8, thus blocking apoptosis [155]. Given that VACV and MCV likely trigger similar innate immune responses, expressing MC159 in the context of VACV is expected to mimic the mechanism by which MC159 would function in the context of an MCV infection. More recently, Huttman et al. utilized murine cytomegalovirus (MCMV) as a surrogate virus to express MC159 [170]. In their study, the authors replaced the MCMV *M36* gene, an MCMV gene that codes for a caspase-8 inhibitor, with MC159. The ΔM36::MC159 virus inhibits TNF-induced apoptosis whereas the ΔM36 MCMV virus does not [170]. Therefore, in the context of surrogate viral infections, MC159 functions similar to what has been reported in ectopic expression studies, at least in terms of apoptotic signaling. However, Huttmann et al. do stress the need for a cell culture system to study MC159, and other MCV immune evasion molecules, in the context of an MCV infection [170].

Like MC159, the MC160 protein contains two tandem death effector domains. However, the 371 amino acid MC160 protein possesses a much longer C-terminus than MC159 [83]. The DEDs of MC160 are 45% and 33% similar to the corresponding DEDs of the MC159 protein [155]. Using the available structure of MC159 protein as a template [166], we recently reported on homology modeling of the MC160 protein [171]. Based on structural alignments between the MC159 and the MC160 DEDs, homology modeling predicts that key hydrogen bonding interactions, such as those present in the charge triads of DED1 and DED2 of MC159, are also present in MC160 [171]. Therefore, the molecular modeling predicts that the overall structures of the MC159 and MC160 DEDs are similar. It should be noted that this prediction will need to be verified with structural studies. Like MC159, MC160 also binds to both FADD and procaspase-8 through MC160 DEDs [147,155]. Unlike MC159, MC160 expression does not appear to affect the extrinsic apoptotic pathways when transfected or expressed in a surrogate VACV [155]. However, there is a single report of anti-apoptotic activity associated with MC160 expression in HEK 293 cells challenged with either Fas or TNF [154]. When MC160 was expressed in a surrogate VACV, MC160 expression failed to prevent the cleavage of caspase-8, -3, or poly[ADP-ribose] polymerase 1(PARP-1) [155]. MC160 also contains caspase cleavage sites and can be a target of caspase-mediated cleavage [155]. Co-expression with MC159 can inhibit caspase-mediated cleavage of MC160 [155]. Interesting parallels can be drawn when comparing MC159/MC160 to cellular cFLIP. Several isoforms of cFLIP exists in cells including the shorter cFLIP_S_ and the long cFLIP_L_. cFLIP_S_ inhibits caspase activation, presumably by associating with procaspase-8 and preventing caspase autoprocessing when cFLIP_S_ inserts into DED oligomers [165]. The longer cFLIP_L_ forms heterodimers with procaspase-8 and promotes caspase-8 activation [172]. Like MC160, cFLIP_L_ is cleaved by procaspase-8 [172]. However, in the case of MC160, independent expression of MC160 does not activate caspase-8 [155]. Interestingly, the caspase-8/cFLIP_L_ heterodimer is only partially active capable of cleaving selected substrates [173]. Therefore, cFLIP_L_ may affect caspase-8 activity in a manner dependent on the concentration of cFLIP_L_, with high concentrations of cFLIPL inhibiting caspase-8 and low concentrations activating [162]. Whether MC160 alters caspase-8 activity in a similar manner remains to be determined. Interestingly, some of the amino acid residues present in MC159 implicated in FADD binding are altered in MC160 relative to MC159 [166]. For example, the MC159 mutant E18A, E19A, and D21A lost the ability to form a ternary complex with Fas/FADD and therefore no longer inhibited apoptosis. Based on structural alignments, equivalent residues in the MC160 DED1 are A16, E17, and D19. Therefore, it is tempting to speculate that MC160 may not bind FADD as strongly as MC159.Whether this variation, and others, throughout the MC160 amino acid sequence accounts for its inability to inhibit apoptosis is not known.

In the context of MCV infection, both MC159 and MC160 are predicted to be expressed concurrently. Therefore, MC160 may play a role in regulating apoptotic responses that is dependent on the co-expression of MC159. The majority of studies focusing on MC159 or MC160 independently express these two viral proteins. Shisler and Moss co-transfected MC159 and MC160 expression vectors, but did not observe enhanced anti-apoptotic effects with cells challenged with Fas antibodies and expressing both MC159 and MC160 relative to cells expressing MC159 alone. Aside from apoptosis, additional functions have been described for both MC159 and MC160.Both MC159 and MC160 inhibit TNFα-induced NF-κB activation and MAVS-mediated induction of IRF-3 and subsequent activation of type I interferons [83,145,146,147,149,150]. MC159 has also been reported to inhibit PKR-induced NF-κB activation and PKR-induced apoptosis [174].

## 3. Poxvirus Inhibitors of the Intrinsic Pathway

### 3.1. Poxvirus Proteins with Bcl-2-Like Folds

Several poxviruses express proteins that adopt a Bcl-2-like fold. VACV has at least eleven proteins with either confirmed or predicted Bcl-2-like structure [11]. Interestingly, these poxvirus proteins share little sequence similarities at the amino acid level to Bcl-2 family member proteins. Despite the pro- and anti-apoptotic roles of cellular Bcl-2 family members, the majority of these poxvirus proteins that share these structural similarities do not actually inhibit apoptosis. Instead, many of these poxvirus proteins have evolved to inhibit cellular innate immune signaling networks [11,82]. For example, Graham et al. demonstrated that VACV proteins A52 and B14 function to inhibit NF-κB activation, despite the presence of the Bcl-2-like fold [175]. Expression of both A52 and B14 in HEK 293 cells dampen NF-κB activation induced by IL-1α. B14 expression, but not A52, prevents activation of NF-κB in response to TNFα as well [175]. Neither A52 nor B14 inhibit apoptosis. The lack of apoptotic inhibition is attributed to missing hydrophobic BH3-peptide binding grooves that are absent in both A52 and B14 [175]. VACV A49 also adopts the Bcl-2 fold, but does not possess the surface groove required to bind BH3 proteins [176]. A49 inhibits NF-κB activation by binding β-transducing repeat containing protein (β-TrCP) as a means to block the ubiquitination of IκBα [177].

Wasilenko et al. originally identified the F1 protein as a novel VACV inhibitor of apoptosis [178]. VACV mutant vv811 expressing F1 is protected from apoptosis induced by staurosporine in Jurkat cells. F1 localizes to the mitochondria via a C-terminal hydrophobic domain where F1 prevents the loss of the mitochondrial membrane potential and subsequent release of cytochrome *c* [178]. Mutant viruses lacking F1L induce apoptosis mediated by Bak/Bax [179,180]. The crystal structure of F1 revealed the characteristic Bcl-2-like fold with affinity for pro-apoptotic proteins with BH3 domains [181]. Several studies have shown F1 interacts with Bim, Bak, and Bax to prevent oligomerization of Bak and Bax and subsequent release of cytochrome *c* into the mitochondria [179,181]. More recently, Campbell et al. reported that F1 achieves its anti-apoptotic function through sequestering Bim [182]. The F1L mutant (A115W) which retains the ability to bind Bak, but not Bim_L_, could not protect cells from mitochondrial mediated apoptosis [182]. In addition, the F1 protein works synergistically with the Vaccinia Growth Factor to counteract infection-induced cell death via a pathway involving Bad [183]. The N-terminus of F1 has also been shown to bind and directly inhibit caspase-9 indicating that F1 may inhibit apoptosis at both the mitochondria and at the level of caspase-9 [184]. In contrast, a recent study by Caria et al. reported that the N-terminal region of F1 is not involved in apoptosis as deletion of the N-terminus does not affect inhibition of apoptosis during a viral infection [185]. However, the F1 N terminus does function as an inhibitor of NLR family pyrin domain containing 1(NLRP1) inflammasome activity during infection [186]. VARV also encodes a homolog of VACV F1. However, unlike VACV F1L, which can inhibit both Bak and Bax-mediated apoptosis, VARV F1 could only block Bax-mediated apoptosis [187].

Unlike the F1 protein, VACV N1 is not located in the mitochondria, but rather is a cytoplasmic dimeric protein [188]. Interestingly, targeting N1 to the mitochondria results in a loss of function suggesting that cytosolic localization of N1 is critical for its anti-apoptotic function [189]. Despite the lack of any Bcl-2 homology at the sequence level, N1 adopts a Bcl-2-like fold with structural similarity to cellular Bcl-X_L_ [190,191]. Cooray et al. showed that N1 expression protects cells from staurosporine induced apoptosis through interaction with pro-apoptotic proteins Bad, Bax, and Bid [190]. However, Postigo and Way found that N1 overexpression did not protect against staurosporine or Bax overexpression induced apoptosis [192]. In addition, when expressed in vv811, N1 did not protect cells against VACV-induced apoptosis compared to either B13 or F1 [125]. Also, infection with the N1L knock out in VACV Western Reserve (WR) does not induce cell death [125,192]. Further, N1L and F1L did not genetically interact, as would be expected from the complementarity of their interacting proteins [192]. Though N1 expression may inhibit the intrinsic apoptotic pathway under certain conditions, the primary function of N1 is thought to be inhibition of NF-κB activation [11]. N1 interacts with both TRAF family member-associated NF-κB activator (TANK) and IKKγ to block NF-κB activation induced by a variety of signals including multiple toll-like receptors, TNFα and IL-1 [193]. Inhibition of NF-κB by the N1 protein is distinct from its ability to block apoptosis. De Motes et al. created separate N1 mutants with mutations in the Bcl-2- like surface groove and mutants in the dimer interface [189]. Mutations in the Bcl-2 groove resulted in N1 mutant proteins that lost the ability to protect cells from apoptosis, whereas mutations in the dimer interface resulted in mutants that could longer inhibit NF-κB activation. Interestingly, only N1 mutants that lost the ability to inhibit NF-κB activation resulted in attenuated VACV infection whereas N1 mutant proteins that could no longer inhibit apoptosis (but still blocked NF-κB) retained virulence in a mouse model [189].

Aside from the orthopoxviruses, several other poxviruses express proteins that adopt a Bcl-2 fold. One of the best characterized proteins is the MYXV M11.M11 is 166 amino acids in length with a C-terminal transmembrane domain that allows M11 to insert into the outer membrane of the mitochondria [194]. The M11L gene is required for virulence. Mutant MYXV with an M11 deletion results in attenuated disease in rabbits with rabbits making a full recovery [195]. M11 inhibits apoptosis induced by multiple challenges including staurosporine and FasL [196,197,198]. The anti-apoptotic activity of M11 was originally linked to M11 association with the peripheral benzodiazepine receptor, a component of the mitochondria permeability transition pore (MPTP) [196]. M11 expression prevents mitochondria membrane permeability and thereby prevents downstream apoptotic effects. However, it was observed that M11 provides protects against mitochondria membrane permeability even in PBR-deficient cells suggesting that M11 uses multiple mechanisms to inhibit apoptosis [196]. Despite lacking sequence homologies to Bcl-2, the crystal structure of M11 identified M11 is a structural mimic of pro-apoptotic Bcl-2 family member proteins [199,200]. M11 interacts with both Bak and Bax and through BH3 domains thereby sequestering these proteins and preventing Bak and Bax oligomerization [197,198,200]. In the case of Bax, M11 does not prevent Bax from translocating to the mitochondria in response to apoptotic signals. Instead, M11 blocks Bax activation at the mitochondria by blocking a Bax conformational change [197].

Since the characterization of N1, F1, and M11, additional poxvirus proteins have been identified with predicted Bcl-2-like folds that inhibit apoptosis. However, identification of such proteins has been difficult as these poxvirus proteins lack sequence homologies to cellular Bcl-2 family members. To circumvent this challenge, Okamoto et al. used the sequence of M11L as the query in a BLASTP search [201]. This approach led to the identification of six additional poxvirus proteins (Deerpox virus DPV022, Swinepox virus SPV12, Shope fibroma virus gp011L, Deer poxvirus DPV022, Lumpy skin disease virus LD17, and Sheeppox virus SPPV14) with similarities to M11 and Bcl-2 family proteins. Of the proteins identified, DPV022, LD17, and SPPV14 significantly prevented cell death in response to ectoposide [201]. Further experiments confirmed that SPPV14 inhibits the intrinsic apoptotic pathway by antagonizing Bak and Bax mediated apoptosis thus preventing the release of cytochrome *c* from the mitochondria to the cytoplasm. Interestingly, SPPV14 can functionally replace F1L and inhibit VACV induced apoptosis when expressed in a VACV with an F1L deletion [201]. Structural analysis of DPV022 identified the Bcl-2 fold [202]. DPV022 inhibits apoptosis through interactions with Bim, Bax, and Bak [202,203].

### 3.2. vGAAP

Camelpoxvirus (CMLV) gene *6L* encodes a hydrophobic protein of 237-amino acids with multiple transmembrane domains [204,205]. This protein was named vGAAP. CMLV vGAAP is expressed early during infection and localizes to the golgi apparatus [204]. To study the virulence of this protein, VACV genomes were screened for equivalent genes. VACV vGAAP is expressed in three strains of VACV (Lister, USSR, and Evans) [204]. The VACV vGAAP expression pattern is identical to that of CMLV and localizes predominantly to the Golgi. Interestingly, homologs of vGAAP are found eukaryotic cells. Both human hGAAP and vGAAP inhibit apoptosis and seem to have overlapping function as vGAAP can complement the loss of hGAAP [204]. hGAAP is 73% identical to vGAAP [204]. When transiently expressed in cells VACV vGAAP inhibits both the intrinsic and extrinsic apoptotic pathways induced by a multitude of challenges including staurosporine, TNFα/cycloheximide (CHX), Fas antibodies, doxorubicin, cisplatin, C2 ceramide, and apoptosis induced by the overexpression of Bax [204]. Interestingly, VACV virus mutants with vGAAP deleted show increased signs of disease and increased viral titers when compared to wild-type and vGAAP revertant virusesin a mouse model [204]. Both vGAAP and hGAAP form ion channels resulting in a passive leak of Ca^2+^ thereby reducing the concentration of Ca^2+^ in the Golgi apparatus [206,207]. Presumably, this leak of Ca^2+^ from intracellular stores affects apoptotic pathways mediated by the release of Ca^2+^. When expressed in cells, vGAAP forms oligomers [206,207]. However, mutant vGAAP proteins that lose the ability to oligomerize retain both anti-apoptotic function and the ability to modulate Ca^2+^ content [207]. vGAAP is the first poxvirus protein identified that forms an ion channel [11,206].

### 3.3. Poxvirus Superoxide Dismutase Homologs

Many poxviruses encode for Cu-Zn superoxide dismutase (SOD) homologs [143,208,209,210,211]. The majority ofthese SOD homologs do not possess enzymatic activity as they are missing critical regions necessary for SOD enzymatic function. One exception is the *Amsactamoorei* entomopoxvirusAMV255 open reading frame which does possess superoxide dismutase activity, though this product is not required for replication [212]. The best studied are the leporipoxvirus SOD homologs present in MYXV and SFV. Neither the M131 nor the S131 proteins possess SOD enzymatic activity [209]. However, leporipoxvirus SOD expression does inhibit the activity of cellular SODS [209]. Both MYXV and SFV Cu-Zn SODs function as decoy proteins by binding copper chaperones for superoxide dismutase (CCS) [211]. Presumably, by leporipoxvirus SODs binding CCS, the levels of active cellular SODs aredecreased. As a result, cellular Cu-Zn SOD is less active and the levels of superoxide increases during infection [210,211]. MYXV M131 expression protects Jurkat cells from apoptosis triggered by both Fas and staurosporine [210]. Conversely, mutant MYXV viruses lacking M131 lose the ability to protect cells from apoptosis [210]. The SFV SOD also contributes to virulence as mutant SFV lacking S131 produce an attenuated infection relative to the parental strain with significantly smaller tumors [210]. Thus, by inhibiting apoptosis, the leporipoxvirus SODs may at least partially be responsible for tumorigenesis observed during infection.

The Molluscum Contagiosum Virus also encodes a Cu/Zn SOD homolog. The MCV SOD homolog, MC163, was originally reported to have homology to sweet potato SOD [143]. Like the SFV proteins, the MC163 protein is predicted to be inactive as it lacks amino acids critical for SOD enzymatic activity. We recently reported that MC163 contains a mitochondrial localization sequence in the N-terminal region [213]. As predicted, MC163 co-localized with pMTurquoise2-mito, a cyan fluorescent protein engineered with a mitochondrial localization sequence (MLS) sequence [213]. Using truncated MC163 mutant proteins, we confirmed that the N-terminal region of MC163 was required for its mitochondria localization. MC163 is the second MCV protein reported to localize to the mitochondria. MC007 was reported to target to the cell’s mitochondria as a means to sequester host retinoblastoma (Rb), which may contribute to dysregulation of the cell cycle during an MCV infection [214]. MC163 expression inhibits apoptosis induced by several challenges including TNF/cycloheximide (CHX) and staurosporine in HeLa cells. Using the 5,5′,6,6′-tetrachloro-1,1′,3,3′-tetraethylbenzimidazolylcarbocyanine iodide (JC-1) mitochondrial membrane potential dye, we demonstrated that MC163 expression prevents mitochondrial membrane permeabilization, an important precursor in the activation of the cell’s intrinsic apoptotic responses [213]. Whether MC163 inhibits apoptosis in a manner similar to the leporipoxvirus SODs by binding CCS as a means to increase reactive oxygen species or functions via a novel mechanism to inhibit apoptosis is the subject of an ongoing investigation.

Though the lack of a tissue culture model has prevented us from studying MC163 during the context of an MCV infection, it is likely that MCV expresses both MC159 and MC163 as a means to further dampen apoptotic signaling events. It is worth noting that MC159 expression could not inhibit staurosporine-induced apoptosis [152], while MC163 expression prevented caspase-3 activation induced by staurosporine [213]. Therefore, MCV may produce MC159 to prevent activation of the extrinsic pathway, while MC163 expression blocks intrinsic apoptosis. In addition to MC159 and MC163, MCV also encodes a third known inhibitor of apoptosis.MC66 was identified as a poxvirus selenocysteine protein with homology to cellular glutathione peroxidases [215]. MC066 expression inhibits apoptosis induced by both UV radiation and hydrogen peroxide. Therefore, during an MCV infection, the anti-apoptotic actions of MC066. MC159, and MC163 along with the ability of MC007 to bind and sequester cellular Rb at the mitochondria likely contribute to the persistence of MCV neoplasms.

The VACV A45R open reading frame has also been identified as a Cu-Zn SOD homolog, conserved in multiple orthopoxviruses [208]. However, whether A45 affects cellular apoptotic pathways is at this time not known. A45 lacks any apparent SOD activity. Interestingly, the 13.5 kDa A45 protein is incorporated into VACV virions [208]. However, the function of A45 in the context of a VACV infection is not well understood. Deletion of the *A45R* gene had no apparent deleterious effect on either VACV replication or virulence in murine or rabbit models [208]. It is possible that the numerous anti-apoptotic proteins produced during a VACV infection could mask the loss of A45. Alternatively, VACV A45 may function differently than what has been reported for MCV and leporipoxvirus SODS.

## 4. Inhibition of dsRNA-Induced Apoptosis

Double stranded RNA is an important pathogen associated molecular pattern (PAMP) produced in cells during viral infection. Though poxviruses possess linear, dsDNA genomes, numerous studies have demonstrated that dsRNA accumulates in the cell during a poxvirus infection [216,217,218,219,220]. Poxviruses do not terminate transcripts efficiently [221]. Therefore, overlapping transcripts of RNA are synthesized from the virus’ RNA polymerase as the enzyme transcribes genes oriented in opposite directions [81,218,220,222,223,224]. This event occurs most frequently during intermediate and late gene transcription. It should be noted, that some dsRNA products can also be detected as a result of overlapping RNAs produced from early gene transcripts [220].

**Figure 2:**
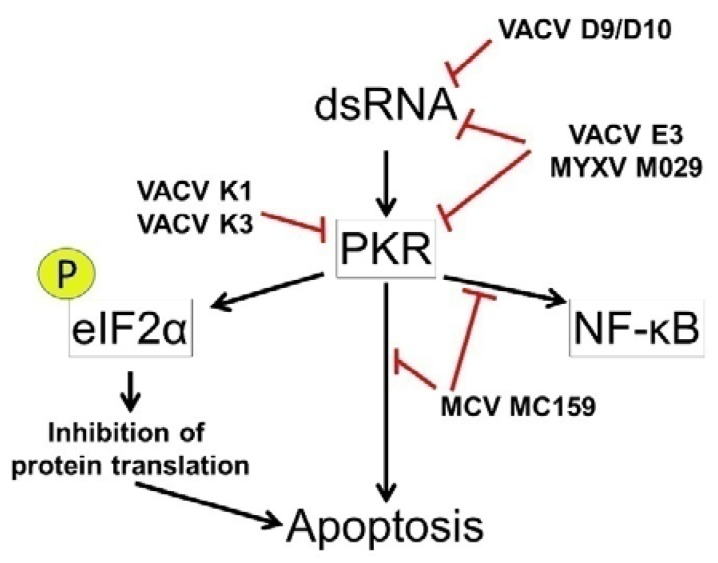
Viral double stranded RNA (dsRNA) activates protein kinase R (PKR). Upon binding dsRNA, PKR becomes activated and elicits several antiviral responses. Active PKR phosphorylates eukaryotic initiation factor 2α (eIF2α) resulting in inhibition of protein translation. PKR can also mediate apoptosis and NF-κB activation. Several poxvirus proteins inhibit PKR activation using a variety of mechanisms. The red lines indicate points in the pathway targeted by several poxvirus immune evasion molecules.

Poxvirus dsRNA induces several innate immune responses including apoptosis [225]. Host cells have several cytoplasmic proteins capable of sensing and responding to viral dsRNA. In the case of poxviruses, one of the best characterized innate immune responses involves activation of protein kinase R. PKR is a serine/threonine kinase that becomes active upon binding dsRNA (Figure 2). Once activated, PKR mediates a variety of antiviral effects including phosphorylation of eIF2α to prevent initiation of protein translation [226]. Activation of PKR also induces the activation of NF-κB, up regulation of type I IFNs, and induction of apoptosis [226,227]. Upon activation, PKR can induce apoptosis through several pathways including one involving FADD and procaspase-8 [227]. Cellular RIG-1 and MDA5 also sense cytoplasmic dsRNA [228,229,230,231,232,233]. These proteins subsequently signal through MAVS complexes culminating in the activation of NF-κB and IRF-3 resulting in the production of type I IFNs [228,234]. In addition to activating IFN, MAVS signaling complexes induce apoptosis [235,236,237,238]. Interestingly, Ferrer et al. reported that Modified Vaccinia Ankara (MVA) and MVAΔFlL viruses induce apoptosis through a pathway at least in part dependent on RIG-I, MDA-5 and MAVS indicating that MAVS signaling towards apoptosis may play a role in poxvirus infected cells [238]. PKR, RIG-1, and MDA5 are all capable of sensing poxvirus dsRNA and inducing antiviral responses [225,238,239,240,241,242,243].

### 4.1. VACV E3

Given the importance of these host pattern recognition receptors (PRRs) in recognizing viral dsRNA to induce an antiviral state, it is not surprising that poxviruses have evolved strategies to counter these host innate immune responses. For example, VACV produces several proteins to mitigate the effects of PKR. The best studied of these proteins is VACV E3. *E3L* is an early gene that encodes for 19 kDa and 25 kDa isoforms due to an alternative AUG initiation site [11,244]. The N-terminal region of the E3 protein contains a Z-DNA binding domain while the C-terminus contains the dsRNA binding domain [245,246,247]. VACV *E3L* is one of several known vaccinia host range genes. Deletion of E3 results in viruses that are incapable of growing in several cell lines including HeLa and Vero, but retain the ability to replicate in BHK21 and CEF cells [225,248,249,250,251,252,253]. While the C-terminal region of E3 is required for replication in HeLa cells, the N-terminal region of E3 is dispensable for replication in most cell lines [248,252]. Though the C-terminal region is required for most of the described functions in cell culture, both the N- and C-terminal regions of E3 are required for pathogenicity in mice models [246,254,255].

The primary role of VACV E3 has been attributed to inhibition of PKR-mediated effects including eIF2α phosphorylation and apoptosis [11,225,245,256,257]. E3L deficient VACV induce PKR activation and apoptosis in HeLa cell lines [225,239]. The importance of PKR in responding to poxvirus infection is highlighted by the observation that replication of E3L deficient viruses is restored when PKR is knocked down in HeLa lines stably expressing small interfering (si)RNA specific for PKR [242]. The most accepted mechanism as to how E3 elicits its effects is that E3 binds viral dsRNA through the C-terminal domain, thus sequestering dsRNA from cellular PRRs [11]. Liu and Moss recently demonstrated that E3 colocalizes with viral factories and dsRNA during VACV infection in A549 cells [258]. Therefore, the E3 protein prevents or reduces the cell’s capabilities to identify viral dsRNA. However, E3 may have additional functions aside from binding dsRNA. Dueck et al. utilized alanine scanning of the dsRNA binding domain to identify E3 mutants with reduced biological function [259]. Of interest, one particular mutant D103A retained the ability to bind polyI:C, a synthetic dsRNA mimic, but could no longer inhibit PKR or block apoptosis as assessed by cleavage of the caspase substrate PARP-1 [259]. In addition to binding dsRNA, E3 physically associates with PKR [260,261]. In light of these observations, the E3 protein may use several mechanisms including binding viral dsRNA as well as novel uncharacterized mechanisms to prevent the activation of PKR. The E3 protein is capable of dampening numerous antiviral pathways mediated by PKR and other cellular PRRs including PKR-induced eIF2α phosphorylation, activation of apoptosis, and activation of NF-κB and type I IFNs [11,262,263,264].

### 4.2. Poxvirus E3 Homologs

E3 is highly conserved in most genera of poxviruses with avipoxviruses and MCV being the notable exceptions. However, the role of these poxvirus E3 homologs play during viral infection has remained largely unexplored. Myskiw et al. expressed E3 homologs from Nigeria goat and sheeppox virus, yaba monkey tumor virus, swinepox, and MYXV in recombinant VACV with E3L deleted [265]. All of the tested E3 homologs bind dsRNA as determined by the ability to immune precipitate with poly I:C. Of the E3 homologs tested, MYXVM029 and swinepox SPV032 displayed the strongest affinity for dsRNA. Interestingly, only MYXV M029 and swinepox SPV032 expressing VACV viruses prevented the activation of PKR and prevented or reduced the cleavage of apoptotic markers caspase-7 and PARP-1 [265]. In addition, when expressed individually in VACV M029 and SPV032 compensate for the loss of E3 and restore growth in the nonpermissive HeLa cell line. However, none of the E3 orthologs restore VACV pathogenicity in mice due to the deletion of E3L [265]. Recently, it has been reported that not all poxviruses produce the same amount of dsRNA during infection. For example, the MPXV produces significantly less dsRNA than VACV, and therefore, may not be as subject to dsRNA mediated responses as VACV [266]. Therefore, E3 homologs from other poxviruses may be able to bind dsRNA with weaker affinity than that of VACV E3, but still contribute to virulence during infection of these virus’ natural hosts. It is also possible that the E3 homologs do contribute to virulence when expressed by their respective viruses using mechanisms similar or different from those employed by VACV E3. Rahman et al. reported that MYXV deleted for M029 is attenuated in susceptible European rabbits and produces no signs of myxomatosis [267]. MYXV *M029L* is also a host range gene with deletion of M029L resulting in mutant viruses that either lose their ability to replicate or grow to lower titers in multiple cell lines including RK13, BSC40, and NIH3T3 [267]. Unlike E3, MYXV M029 lacks the N-terminal region required for binding Z DNA [268]. Similar to observations reported regarding E3L, deletion of M029L from MYXV results in a virus that upregulates activates PKR [267]. When E3L is stably expressed in the RK13 cell lines, M029L deleted viruses grow as efficiently as wild-type, further highlighting that M029 and E3 proteins have similar functions [267]. Similar to observations with E3 deletions, knocking down PKR restores replication in M029 deleted viruses [267]. M029 also immunopreciptates with PKR in an association that is dependent on the presence of dsRNA [267].

### 4.3. Poxvirus Decapping Enzymes

In addition to E3 and E3 homologs, poxviruses encode multiple proteins to inhibit the effects of dsRNA-induced PKR activation. VACV alone has at least four proteins (K1, C7, E3, and K3) that block inhibit PKR activation by various mechanisms [82,269]. Recently, the VACV decapping enzymes D9 and D10 were reported to prevent activation of PKR as well [217,270]. D9 and D10 prevent the accumulation of dsRNA by decapping viral mRNAs. These decapped viral mRNAs are then subject to degradation by the host exonuclease Xrn1 [217]. Transfecting cells with siRNA specific to Xrn1 results in accumulation of viral dsRNA and subsequent activation of both PKR and the cellular RNaseL pathways during VACV infection [217]. Further, siRNA targeting Xrn1 significantly reduces viral titers. Interestingly, E3 was well expressed even when Xrn1 was depleted. Therefore, the data presented by Burgess and Mohr suggest that E3 can become overwhelmed by excess amounts of dsRNA that accumulate in the absence of Xrn1 [217]. However, VACV with an E3L deletioninduce PKR and eIF2α phosphorylation to higher amounts and at earlier times than what is observed in viruses lacking functional decapping enzymes [258]. This observation correlated with much less viral protein production in vΔE3L viruses than what was observed in vD9muD10mu viruses. Further, D9 and D10 are not detected in viruses with E3 deleted [258]. Therefore, both E3 and the decapping enzymes are necessary to prevent dsRNA induced anti-viral pathways.

Liu et al. further demonstrated that catalytic site mutations in D9 and D10 result in a mutant VACV with reduced replication kinetics in BS-C-1 and HeLa cells [270]. The vD9muD10mu VACV activate PKR and RNaseL in BS-C-1 cells due to increased levels of dsRNA produced during infection. Interestingly, the E3 present in the vD9muD10mu VACV is not sufficient to block PKR activation in the absence of active D9 and D10 [217,270]. Presumably, this increase in levels of dsRNA could also trigger apoptotic pathways through PKR, thereby preventing virus infection. Utilizing clustered regularly interspaced short palindromic repeats-CRISPR associated protein 9 (CRISPR-Cas9) to create double knockouts of PKR and RNase L as well as triple knockouts of Xrn1, PKR, and RNase L, Liu and Moss demonstrated that in the absence of PKR and RNase L, protein synthesis is restored in vD9muD10mu as well as vΔE3L viruses [258]. However, while vΔE3L viral titers were restored in double and triple knockout cells to that of the control virus expressing E3 and decapping enzymes in control cells, the vD9muD10mu titers remained low in double and triple knockout A549 cells suggesting additional inhibitory mechanisms may be in play [258]. By expressing both E3 and the decapping enzymes, VACV ensures that the levels of dsRNA are kept at concentrations below the threshold necessary to trigger the cells innate immune response to dsRNA [217,258,270].

### 4.4. MCV MC159 Inhibits PKR-Induced Apoptosis

Responses to dsRNA in MCV have not been well characterized. The replication and transcription processes of MCV and VACV are expected to be similar, and therefore, MCV likely produces dsRNA during infection. However, due to the lack of a suitable cell culture model to study MCV, MCV dsRNA production during an MCV infection has never been demonstrated. In addition, the activation state of PKR during an MCV infection has yet to be determined. PKR is well expressed in keratinocytes and capable of sensing dsRNA [271]. Therefore, it seems reasonable to speculate that MCV must counteract the effects of PKR to cause a persistent infection. MCV lacks an E3 homolog to bind any MCV dsRNA that may be produced during infection [142,143]. A single study by Gil et al. reported that the MC159 protein blocks PKR-induced activation of apoptosis in HeLa cells when MC159 and PKR are each expressed from a recombinant VACV [174]. MC159 expression also blocks PKR-induced NF-κB activation. However, MC159 could not prevent the phosphorylation of eIF2α and could not overcome the additional antiviral effects of PKR [174]. Therefore, how MCV inhibits the antiviral effects of PKR remain largely unknown. Like VACV, MCV also encodes decapping enzymes (MC098 and MC099) [142,143]. Whether these MCV products have a similar function as the aforementioned VACV enzymes remains to be determined.

## 5. Apoptotic Mimicry

During apoptosis, the inner leaflet of the cell membrane becomes reversed exposing phosphatidylserine (PS) on the outside of apoptotic bodies. The now exposed PS serves as a signal for phagocytes to clear the apoptotic bodies. This process induces a strong anti-inflammatory signal. Therefore, apoptotic cells, unlike necrotic cells, do not induce an inflammatory response [272].

Vaccinia virus mature virions (MV) contain PS in the viral envelopes [273,274,275]. The PS present in the VACV envelope is likely derived from the PS-rich ER luminal leaflet [272,276,277,278,279]. Mercer and Helenius reported that VACV utilizes macropinocytosis as a means to enter cells via a process dependent on the presence of PS [280]. Laliberte and Moss further demonstrated that purified VACV MVs extracted with NP-40 detergent could be reconstituted with PS resulting in restoration of infectivity [281]. As this method of viral entrance is similar to the uptake of apoptotic bodies, the term apoptotic mimicry is used to refer to virus entry that use similar methods dependent on PS to gain entry into the cells [272]. Indeed, VACV is one of several enveloped viruses to utilize this mechanism to gain entry into host cells (Reviewed in [272]). Essentially, VACV mimics an apoptotic body to trick the cell into ingesting VACV. Blebbistatin, an inhibitor of micropinocytosis, prevents entry of VACV WR, thus confirming a role for macropinocytosis in poxvirus entry [280,281]. Both forms of VACV virions, MV and extracellular virions (EV), use macropinocytosisas a means to gain entry into host cells [85,280,282,283,284,285]. The soluble serum protein Gas6 servers as a bridge interacting with both PS in the VACV envelope and the cellular TAM receptor Axl [286]. Gas6 significantly enhances the entrance of EV in HMEC cells.Further, anti-Axl antibodies reduce the ability of VACV to infect HMECs. Conversely, HEK 293T cells overexpressing Axl enhance VACV EV infectivity [286].

Given that apoptotic clearing is associated with dampening inflammatory responses, it is possible that VACV and other poxviruses utilize apoptotic mimicry as a means to subvert immune response [272]. For example, transcription of toll-like receptor and cytokine suppressor molecules SOCS1 and SOCS3 (suppressor of cytokine signaling 1 and 3) is enhanced upon PS-Gas6 binding to TAM receptors [85,287]. In general, virus interaction with TAM receptors is thought to play a role in suppression of innate immune responses. Bhattacharyya et al. used pseudotyped human immunodeficiency virus 1 (HIV-1)-derived viruses to demonstrate minimal upregulation of type I IFN mRNAs and upregulation of SOCS1 mRNA in wild-type bone-marrow-derived dendritic cells (BMDCs) [288]. However, in TAM knockout cells, infection resulted in a significant upregulation of type I IFN mRNAs. Therefore, virus-induced activation of TAM receptors represents a means to prevent type I IFN expression [288]. During apoptotic clearance, transforming growth factor-β (TGF-β) and IL-10 are produced to prevent inflammatory responses [85,272,289,290]. Interestingly, VACV infection also induces anti-inflammatory cytokines [85,272,291,292]. Therefore, VACV and other poxviruses may utilize apoptotic mimicry as a general means to suppress host immune responses by taking advantage of cellular mechanisms to clear apoptotic bodies. In the case of MCV, NI-MC lesions are almost completely undetected by immune cells [144]. Therefore, it is tempting to speculate that VACV, MCV, and other poxviruses might use apoptotic mimicry as a means to remain undetected by the immune system [85,272]. If apoptotic mimicry does play a role in poxvirus immune suppression, this mechanism could delay poxvirus detection, thereby allowing the virus to spread and cause persistent infections.

## 6. Conclusions

Multiple poxvirus proteins from several genera have been described to date that inhibit apoptosis. The study of the molecular mechanisms by which these proteins elicit their anti-apoptotic function has led to a better understanding of how these viruses have adapted to survive in host cells. In addition, through the study of poxvirus anti-apoptotic proteins, researchers have gained a better understanding of how host cells detect and respond to virus infection. This knowledge is fundamentally important as poxviruses continue to be evaluated for use as vaccine vectors and oncolytic viruses. Given the large size of poxvirus genomes, it is important to note that additional viral products that antagonize cell death may be present and have yet to be discovered.

## Figures and Tables

**Table 1:** Summary of poxvirus products with anti-apoptotic function.

Protein	Type of Protein	Virus	Function(s)
CrmA	Serpin	CPXV	Inhibits caspase(s) activity
			Reduces inflammation and promotes viral replication
B13 (SPI-2)	Serpin	VACV	Inhibits caspase(s) activity
B22 (SPI-1)	Serpin	VACV	Inhibits caspase(s) activity
SPI-3	Serpin	VACV	Inhibits caspase(s) activity. Inhibits cell fusion
SERP1	Serpin	MYXV	Inhibits caspase(s) activity
			Provides full virulence
			Reduces inflammation
SERP2	Serpin	MYXV	Inhibits caspase(s) activity
			Involved in lesion morphology
			Promotes myxomatosis
SERP3	Serpin	MYXV	Inhibits caspase(s) activity
			Involved in lesion morphology
CrmB	vTNFR	VACV	Mimics extracellular domain of TNFR1/2
			Enhances virulence
CrmC	vTNFR	VACV	Mimics extracellular domain of TNFR1/2
			Enhances virulence
CrmD	vTNFR	ECTV	Mimics extracellular domain of TNFR1/2
			Possesses SECRET domain that binds to chemokines
CrmE	vTNFR	VACV	Mimics extracellular domain of TNFR1/2
			Enhances virulence
M-T2	vTNFR	MYXV	Mimics extracellular domain of TNFR1/2.
			Secreted form inhibits TNF
			Intracellular form blocks virus induced lymphocyte apoptosis
T2	vTNFR	SFV	Mimics extracellular domain of TNFR1/2
			Inhibits cellular TNF
A52	Bcl-2-like folds	VACV	Inhibits IL-1 induced NF-κB activation
B14	Bcl-2-like folds	VACV	Inhibits IL-1 induced NF-κB activation
A49	Bcl-2-like folds	VACV	Inhibits NF-κB activation through interactions with β-TrCP
F1	Bcl-2-like folds	VACV	Inhibits staurosporine induced apoptosis
			Localizes to the mitochondria
N1	Bcl-2-like folds	VACV	Inhibits staurosporine induced apoptosis
			Interacts with Bad, Bax and Bid
			Inhibits NF-κB activation
			Localizes in cytosol
M11	Bcl-2-like folds	MYXV	Required for virulence
			Inhibits FasL and staurosporine induced apoptosis
			Interacts with Bak and Bax
DPV022	Bcl-2-like folds	DPV	Inhibits apoptosis induced by Bax and Bak Interacts with Bim, Bax, and Bak
SPPV14	Bcl-2-like folds	SPPV14	Inhibits intrinsic apoptosis by antagonizing Bak and Bax
6L	vGAAP	CMLV	Inhibits extrinsic and intrinsic apoptosis
			Forms ion channels reducing concentration of Ca^2+^ in golgi apparatus
M131	SOD Homolog	MYXV	Binds copper chaperones for superoxide dismutase (CCS)
			Cellular Cu-Zn SOD less active resulting in increased superoxide levels
			Protects cells from apoptosis
S131	SOD Homolog	SFV	Binds CCS
			Cellular Cu-Zn SOD less active resulting in increased superoxide levels
			Protects cells from apoptosis
			Aids in virulence.
MC163	SOD Homolog	MCV	Inhibits TNFα-induced apoptosis by preventing MMP.
			Localizes to the mitochondria
			Prevents staurosporine induced caspase 3 activation
MC066	Seleoncystein protein	MCV	Inhibits UV and hydrogen peroxide induced apoptosis
A45	SOD Homolog	VACV	Function currently unknown
E3	PKR antagonist	VACV	Inhibits PKR activation by sequestering dsRNA
			Binds to PKR
			Required for virulence
M029	E3 homolog	MYXV	Inhibits PKR activation
			Reduces/prevents cleavage of caspase-7 and PARP-1
SPV032	E3 homolog	SPV	Inhibits PKR activation
			Reduces/prevents cleavage of caspase-7 and PARP-1
D9/D10	Decapping enzymes	VACV	Inhibits PKR activation by reducing dsRNA accumulation
MC159	vFLIP	MCV	Inhibits TNFα and FasL induced apoptosis
			Interacts with FADD and procaspase-8
			Prevent caspase 3 and caspase 8 activation Inhibits TNFα induced NFκB activation and MAVS-induced IRF-3 activation
MC160	vFLIP	MCV	Inhibits TNFα induced NFκB activation and MAVS-
			induced IRF-3 activation

Abbreviations: CPXV, Cowpox Virus; VACV, Vaccinia Virus; MYXV, Myxoma Virus; ECTV, Ectromelia Virus; SFV, Shope Fibroma Virus; MCV, Molluscum Contagiosum Virus; SPV. Swinepox Virus; CMLV, Camelpox Virus; DPV, Deerpox Virus; vTNFR, viral tumor necrosis factor receptor; vGAAP, viral Golgi anti-apoptotic protein; PKR, protein kinase R; vFLIP, viral FLICE inhibitory protein; SOD, superoxide dismutase; IL-1, interleukin-1; β-TrCP, β-transducing repeat containing protein; Bad, Bcl-2 associated death promoter; Bcl-associated X protein; Bid, BH3 interacting-domain death agonist; NF-κB, nuclear factor κ B; Bak, Bcl-2 homologous antagonist killer; Bim, Bcl-2-like protein 11; TNFα, tumor necrosis factor-α; MMP, mitochondrial membrane permeabilization; UV, ultraviolet; PARP-1, poly (ADP-ribose) polymerase 1; MAVS, mitochondrial antiviral-signaling protein; IRF-3, interferon regulatory transcription factor 3.
